# Virtual reality in pain therapy: a requirements analysis for older adults with chronic back pain

**DOI:** 10.1186/s12984-020-00753-8

**Published:** 2020-09-29

**Authors:** Oskar Stamm, Rebecca Dahms, Ursula Müller-Werdan

**Affiliations:** Geriatrics Research Group - Age and Technology Charité - Universitätsmedizin Berlin, corporate member of Freie Universität Berlin, Humboldt-Universität zu Berlin, and Berlin Institute of Health, Reinickendorfer Strasse 61, 13347 Berlin, Germany

**Keywords:** Chronic Back pain, Virtual reality treatment program, Exergame, Geriatrics, Physiotherapy, Psychotherapy

## Abstract

**Background:**

Today immersive environments such as Virtual Reality (VR) offer new opportunities for serious gaming in exercise therapy and psychoeducation. Chronic back pain (CBP) patients could benefit from exergames in VR. The requirements in older CBP patients for a VR pain therapy have not yet been determined in studies. The aim of the study was to perform a requirements analysis for the user group of geriatric patients with CBP for a VR exergame. The objective was to find out the expectations, desires, preferences and barriers in order to collect them as requirements for this vulnerable group and to determine frameworks of therapy by physiotherapists and psychotherapists.

**Methods:**

We conducted a requirements analysis through semi-structured interviews with 10 elderly participants with CBP. Furthermore, two focus groups were conducted with three physiotherapists and two psychotherapists to determine frameworks of therapy programs for the target group. The qualitative data were transcribed and examined through a structuring content analysis. Subsequently, the results of the analysis were prioritized by all participants of the study.

**Results:**

The results of the requirements analysis indicate mandatory requirements for the overall system, hardware, software and gamification elements. The key requirements were target-group-specific applications of the VR exergame through e.g. individual briefing, user-friendly handling, inclusion of movement limitations, presentation of everyday scenarios in combination with biofeedback, age-appropriate feedback through praise and awards and a maximum exercise duration of 30 min and 15 min of relaxation.

**Conclusion:**

It should be possible to use the determined requirements productively to create user-friendly VR exergames that motivate elderly chronic back pain patients to perform exercises regularly.

**Trial registration:**

The study is registered in the German Clinical Trials Register (DRKS-ID: DRKS00015294 12.10.2018).

## Introduction

### Background

Back pain is the most common musculoskeletal condition and a major health problem. Back pain can be acute, when it lasts for less than 4 weeks, or subacute, when the pain lasts between 4 weeks and 3 months. If the pain persists for more than 12 to 24 weeks, a chronic course is assumed [1]. Although back pain is often considered a job-related condition, it is also a major disabling health condition among older adults. More than 50% of Europeans over the age of 75 suffer from moderate to severe pain [2]. In Germany the highest prevalence of chronic back pain (CBP) in women and men is in the age group over 70 years old [3].

CBP has a multifactorial etiology and patients have often kinesiophobia, fear avoidance beliefs and passive coping strategies, which can lead to illness behavior and physical disability [4]. Since psychosocial factors in particular are crucial for a chronic development of back pain, it is important to include the patients’ beliefs and coping strategies in addition to the physical treatment. Readjusting the developed beliefs by learning new strategies leads to a change of behavior and to psychological changes. Therefore, CBP is often treated by multimodal pain therapy, usually consisting of medical, physiotherapeutic, physical and psychotherapeutic pain therapy. Several studies have shown that multimodal pain therapy has greater effectiveness than conventional treatment e.g. for improving pain intensity and increasing physical function [5].

One of the most important factors for the treatment of CBP is the adherence to regularly performed home exercises. An effective therapy requires a high degree of initiative by the patients, which can be an obstacle for many. Studies in physiotherapy indicate that patients who did not adhere to exercises had less treatment effect [6–8]. In the course of multimodal pain therapy, the patient learns exercises helping him or her to reduce pain even after completion of the therapy. However, home exercises are too rarely performed by the patients. Slujis et al. [9], showed that only 35% of the instructed subjects (*N* = 1178) performed exercises at home. Similar results were shown by Göhner et al. [10]. So-called exergames, which are games that combine exercises and gameplay could decrease the non-adherence for prescribed home exercises and motivate patient to a long-term use with a higher training frequency. Thus, gamification elements can be used specifically as encouragement in a targeted manner for patients. First studies in this field indicated that a specially developed Nintendo Wii exergame could be a biopsychosocial intervention for chronic low back pain [11, 12]. Furthermore, the Microsoft Kinect was utilized in a study [13], which compared a group receiving an exergame treatment with a conventional treatment group. The study showed a significant improvement in muscle strength in the subjects of the exergame group in contrast to the conventional training group.

Immersive environments such as Virtual Reality (VR) nowadays offer new opportunities for serious gaming in exercise therapy. The most popular VR game of the year 2019 according to Steam’s Top Sellers List as measured by gross revenue was the rhythm game Beat Saber, which was the only VR title on the list. Kivelä et al. showed that VR games can be played as physical work out. In their study it was shown that the heart rate increases by playing Beat Saber [14]. Laver et al. [15] displayed in their systematic review about VR for stroke rehabilitation, that VR may be beneficial in improving upper limb function and activities of daily living function when used as an adjunct to usual care. Jones et al. [16] showed the impact of a low-motion VR game application on chronic pain. The pain of the participants in this study was reduced after the VR treatment by 33%. Tashjian et al. [17] indicated similar results in their study comparing 3D VR and a 2D video for the purposes of pain reduction, finding that the pain reduction was greater in the VR cohort than in the control group. These results show that VR can be utilized for pain management. Appel et al. demonstrated that it is feasible and safe to expose older adults with various levels of cognitive and physical impairments to immersive VR [18]. However, there are hardly any studies or applications on the market offering a VR active exercise therapy and psychotherapeutic pain therapy beyond the pure VR pain distraction applications. Furthermore, there are no studies investigating an active VR exergame with an HMD for older chronic back pain patients. The review of Skjæret [19], which included 60 exergame studies, revealed that the Nintendo Wii console was the most frequently used console in exergame studies and measures of balance were the most frequently used outcome measures (e.g. [20–22]). In order to secure an effective long-term therapy for chronic back pain patients, a multimodal approach in the VR is necessary in the future.

Within the scope of the ViRST project, we plan not only to determine measurements for various physiological outcomes, but also offer psychological exercises and behavioral recommendations to manage fear avoidance beliefs or poor stress management during the back pain treatment training. In this context, the aim of our study was to explore the question: what are the requirements for an exergame, which could be used to supplement the multimodal pain therapy to increase the adherence of CBP patients. Therefore, we wanted to find out the expectations, desires, preferences and barriers of the patients on one hand and determine frameworks of therapy by physiotherapists and psychotherapists on the other hand.

## Methods

### General

The target group of geriatric patients often has difficulties accessing existing technology, which can lead to the exclusion of this vulnerable group from some technologies. For this reason, a user-centered design is indispensable for older chronic back pain patients. In order to create user-friendly, adaptive, personalized system that is tailored to the target group, we conducted a requirements analysis as a first step.

The study had an explorative, qualitative approach. In addition, quantitative assessments were only used for gathering baseline data from the CBP. The study consisted of two focus group interviews with three physiotherapists and with two psychotherapists and semi-structured interviews with 10 geriatric CBP patients. The purpose was to find out the expectations, desires, preferences and barriers of this vulnerable group and the experts. The study protocol was approved by the ethics committee and data protection committee of the Charité. All participants gave their written informed consent to take part in the interviews.

### Procedure

#### Interviews with older CBP patients

The purpose of first part of the qualitative study was to assess the requirements of a VR exergame for geriatric patients with CBP. The sample (*n* = 10) consisted of older adults over 65 years with diagnosed chronic back pain. Interested persons were first informed about the study in a telephone call and then, if accepted, asked about inclusion and exclusion criteria in a screening. After initial screening and formal inclusion, the participants completed an informed consent form, the SF-12 Health Survey (Short Form 12 German translation) and the chronic pain grade questionnaire (CPGQ). Subsequently semi-structured interviews were conducted in order to investigate the requirements of the seniors. The semi-structured interview guideline created by the Geriatrics Research Group included the following main content categories:

- sports and gymnastics practiced in the past and today,
- sport as social participation,
- problems and difficulties in activities of daily life,
- strategies for dealing with pain,
- game experiences and preferences (traditional and digital games),
- hardware and software requirements for a VR exergame
- requirements for motivational elements.

The interviews with the CBP patients lasted 40–60 min and were divided into three parts; a first part for personal requirements, a second in which the subjects tested a user experience demo in a VR environment, and a third concerning the requirements for a VR pain therapy system. Afterwards the interviews were transcribed and analyzed. The Ethics Committee of the Charité approved the study protocol (no. of approval: EA4/055/18).

#### Focus group

The second part of the qualitative study included two focus groups. The intention was to gather framework data and requirements from physiotherapists (*n* = 3) and psychotherapists (*n* = 2) who have expertise in older CBP patients. The partly standardized focus group interviews were carried out with guidelines, which included questions on pain management experiences and games applications in therapy. The first part of the guidelines included the following sub-topics:

- Experience in the treatment of chronic pain patients,
- adherence of patients (physiotherapists) / overcoming the fear-avoidance behavior (psychotherapists),
- acceptance and experience with game applications in pain therapy.

Subsequently, a short film sequence about Virtual Reality Therapy was presented to show already existing concepts. The second part of the guidelines included questions about requirements for a VR therapy system with the following sub-topics:

- Software (patient’s UI), software (therapist’s UI),
- hardware,
- and communication with therapists / forwarding of data.

The survey in the focus groups lasted 1–1.5 h.

### Materials

#### Assessments

The SF-12 Health Survey tested on older adults was found to be reliable and valid [23]. Likewise, the chronic pain grade questionnaire is a valid and reliable tool. The internal consistency shown with Cronbach’s alpha = 0.74 for chronic back pain was determined by Korff et al. [24]. The study of Penny et al. [25] had shown a relationship between the CPGQ score and the Short Form 36 Health Survey (SF-36). The results of the study confirm that a higher chronic pain grade is associated with poorer health in all aspects such as poorer physical, psychological, and social health, which supports a multimodal pain management approach.

#### VR headset

During the task-based part of the semi-structured interviews the seniors and experts tested two applications on the Dell Visor VR118 Headset. This headset has a 105-degree horizontal field of view and a display rate up to 90 Hz. This VR headset was chosen because of the two high-resolution displays at 1440 × 1440, so that the testing group was not exposed to cyber sickness.

#### VR apps

**Fig. 1:**
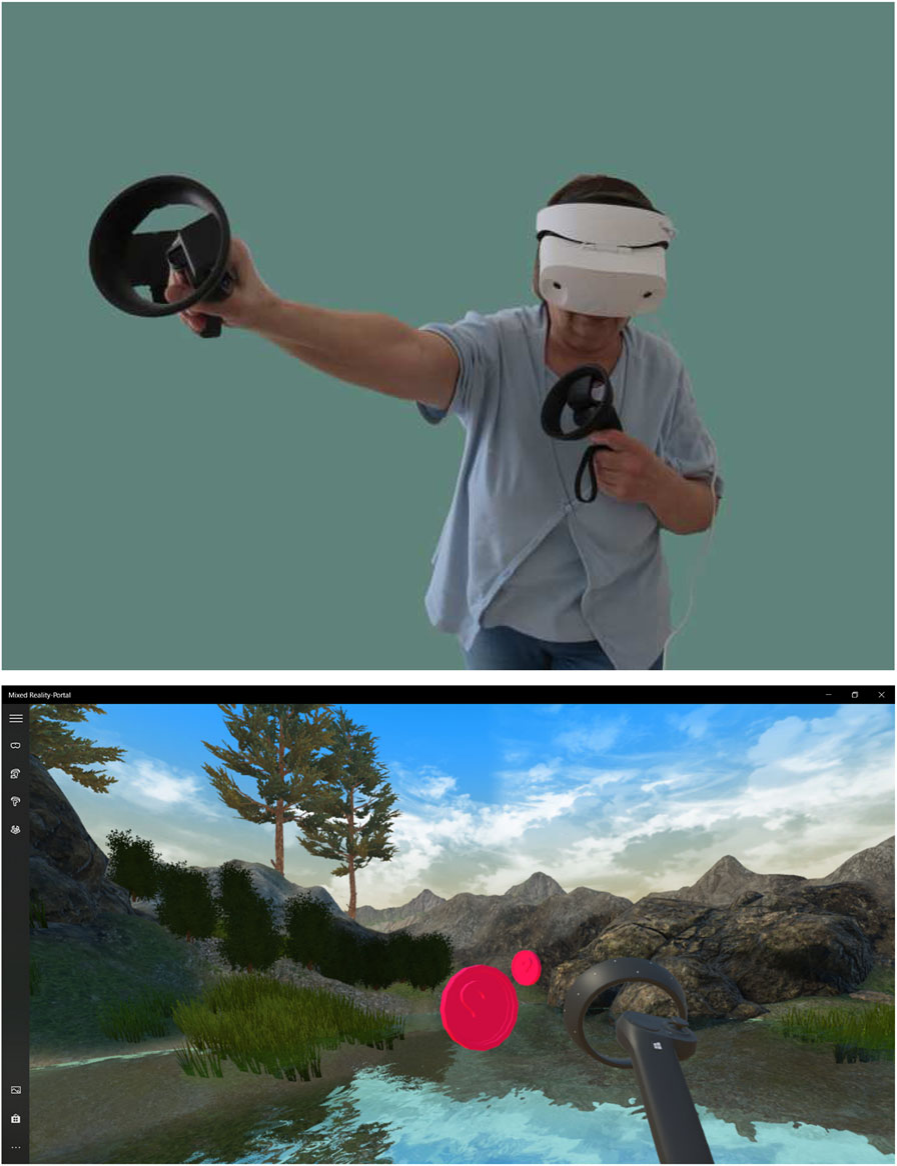
VR experience prototype

In order to create a common understanding for VR in the sample of seniors an exergame user experience prototype was used (Fig. 1). In the prototype, the player stands on a platform on a lake and has to collect pink coins. These appear for a short time around the player. The player’s straight posture is determined by the headset height at the beginning of the application, and subsequent malposition of the back turns the player’s surroundings from a colorful landscape to black and white one. The software prototype did not contain any other gamification aspects, e.g. a story, in order not to influence the user’s requirements on this topic.

The second application was the HoloTour by Microsoft Corporation. The aim was that the player experiences the feeling of immersion in a realistic-looking virtual space.

### Data analysis

The interviews with the seniors were recorded and subsequently transcribed using the transcription software f4. A summarizing content analysis by Mayring (Mayring 2015) was applied for the data analysis. As an analyzing tool, we used the qualitative data analysis software Atlas.ti 8. The structuring content analysis contains paraphrasing, generalization to abstraction level and reduction. The transcripts were coded manually according to specially drafted coding rules. The coding was carried out by two scientists, using the four-eyes principle to ensure reliable coding. The coding and analysis were controlled reciprocally. A total of 130 codes were assigned and 1329 quotations were classified as relevant.

### Participants

#### Chronic back pain patients

Ten participants with CBP were included in the semi-structured interviews. One subject had to be excluded from the study. The inclusion criteria were:

- Geriatric patients with chronic back pain longer than 6 months
- ≥ 65 years
- No cognitive impairments
- Independent mobility
- Able to perform exercises actively
- No spinal malignancies, spondylitis and spondylodiscitis, fibromyalgia
- No disc surgery in his or her medical history
- No strong vestibular disturbances that affect balance ability

The exclusion criteria were defined as:

- Patients with chronic back pain shorter than 6 months
- < 65 years
- Cognitive impairments
- Immobility or mobility only possible with help
- Sensory and / or motor impairments
- Unable to do exercises actively
- Spinal malignancies, spondylitis and spondylodiscitis, fibromyalgia
- Disc surgery in his or her medical history
- Strong vestibular disturbances that affect balance ability

On average the CBP patients were 75.9 years (SD 6.9) old. Seven participants of the sample had a low disability and a low intensity of pain, corresponding to Grade I in Von Korff’s chronic pain scale [24]. The pain severity of one participant was classified as Grade II, which means: low disability and high intensity. Two participants had a high disability, which is moderately limiting and corresponds to Grade III. None of the probands’ pain was classified as Grade IV with a high disability, which is severely limiting. All participants had back pain for more than 6 months and were able to perform exercises actively. None of the subjects had cognitive limitations or severe balance limitations. The subjects had in the Short Form (12) Health Survey, which is a shorter version of the SF-36, a mean mental health score of 53 (SD 8.0) and a physical health score of 37 (SD 10.7) on a scale from 0 to 100. Both mean values of the SF-12 ​​are slightly higher than the comparative values of patients with lumbar back pain (mental health: 47.3 and physical health: 35.1) [26]. In a questionnaire for the subjective evaluation of technology use, the majority of subjects (*n* = 6) stated that they frequently use their technology devices. All subjects stated that they use a telephone and a radio. As VR still requires a PC or at least a smartphone, the question of PC and smartphone usage has been an important clue to starting conditions in the sample. Eight subjects stated that they use a PC and six that they use a smartphone.

#### Experts

As physiotherapists and psychotherapists are an important part of multimodal treatment teams, they have been included as experts concerning the requirements of a potential VR exergame. The inclusion criteria were:

- Experience in the treatment of chronic back pain patients
- at least 3 years professional experience with back pain patients
- Currently working as physiotherapist or psychotherapist

Two focus group interviews were conducted, one with three physiotherapists and one with two psychotherapists. Among the physiotherapists, two of the participants are employed in a geriatric rehabilitation clinic and one in a physiotherapy center. The professional experience among the physiotherapists was between 4 and 10 years. The second focus group was attended by two psychotherapists, one of whom works in a clinic with focus on acute geriatric medicine and one who owns a psychotherapy practice. The professional experience of the participants was 6.5 and 14 years.

### Prioritization of the requirements

**Fig. 2:**
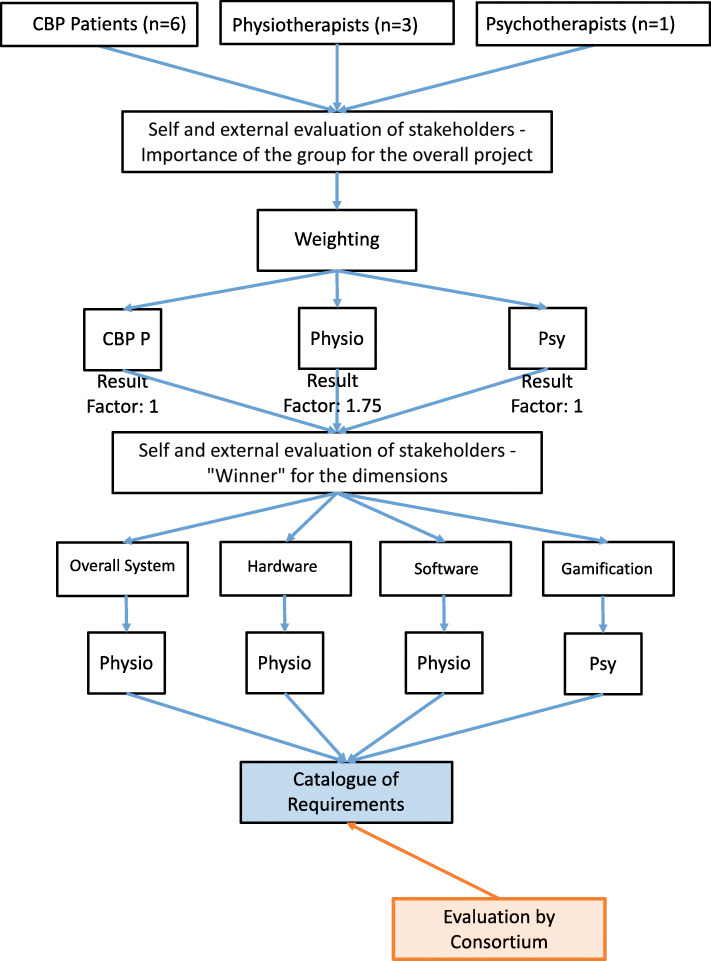
Prioritization procedure based on Moisiadis’ prioritization method (Moisiadis, 2002) of self and external evaluation of stakeholder profiles

The requirements analysis summarizes the requirements determined in a catalogue, which is the basis for the conception of the joint project and the further structuring of the VR training program. In order to create a common understanding among the stakeholders about the importance of the requirements, a prioritization was carried out. Following the content analysis, the reduced requirements of the semi-structured interviews and the focus groups were prioritized by six CBP patients, three physiotherapists and one psychotherapist. The prioritization procedure (Fig. 2) was based on Moisiadis’ prioritization method (Moisiadis, 2002). The prioritization aimed at a self-prioritization and external prioritization of the requirements by various stakeholders. Rankings of 1 to 3 were used to determine the rankings of the three stakeholders (1 = CBP patients, 2 = physiotherapists, 3 = psychotherapists) for the overall project and for each of the four dimensions (overall system, hardware, software, gamification). A weighting for the requirements was calculated from the rankings for the overall project. The requirements in the four dimensions were also ranked according to their subjective importance and calculated with the weighted stakeholder factor. Within the stakeholder groups, mean values were used to determine the average stakeholder ranking for the requirements, resulting in a ranking of the requirements per stakeholder that could be compared between the stakeholders. The stakeholder rankings in the dimensions made it possible to determine a “winner stakeholder” for each dimension. The requirements of these stakeholders were subsequently included in the catalog of requirements for each dimension and evaluated again by the consortium using the MoSCoW prioritizations [27] according to the project relevance.

### Weighting of the requirements

In order to evaluate the importance of the opinion of the expert groups involved (CBP patients, physiotherapists, psychotherapists) for the overall project, the stakeholders were rated. Profile models similar to Moisiadis’ prioritization method were created for the stakeholders. As part of the prioritization process, the stakeholders were assessed by the participants using subjective and objective ratings. All participants were involved in advance in the interviews to determine the requirements. The rankings were subsequently decoded, with 3rd place receiving 0 points, 2nd place 50 points and 1st place 100 points. Afterwards a weighting was calculated from the mean values of the scores. The evaluation showed that the physiotherapists achieved 1st place and the psychotherapists and CBP patients achieved 2nd place together. The physiotherapists received a 1.75 times higher rating than both other stakeholders, which resulted in a factor of 1.75 for further rating of the four dimensions. The further dimensions, which were ranked on a weighted basis in addition to the overall project, were: overall system, hardware, software and gamification. The categories will be presented in the following.

## Results

In the following, the results for the identified dimensions from the summarizing content analysis of the interviews with the CBP patients and the focus groups with the physiotherapists and psychotherapists will be presented. The dimensions consist of semantic levels, which resulted from the clustering of the content analysis and represent the code groups that emerged. Each level consists of the requirements that were gathered from interviews with all stakeholders. After decoding of the requirements rankings (transformation of rankings into percentiles) we were able to calculate average values within the stakeholder groups for the requirements. The rating of each stakeholder’s opinion for each dimension made it possible to identify a “winning stakeholder”. Each semantic level of the dimensions is presented in the following by the requirements that were prioritized as the most important.

### Overall system

This dimension consists of the following levels: *briefing/instruction, handling, duration of use, safety and price/financing*. In the dimension *overall system*, the participants identified the physiotherapists as the most important stakeholder. The psychotherapists were ranked 2nd and the CBP patients 3rd. The ranking of the requirements of physiotherapists were taken over for this dimension.

The physiotherapists considered the requirement: **The system should offer an individual briefing** as the most important requirement in the semantic level *briefing/instruction*. There are several ways that the system could be made usable for geriatric chronic back pain patients. **The system should offer a demo (tutorial) for correct operation** was prioritized as the second most important requirement. **The briefing should be carried out by personal assistance was prioritized** with rank three. Both of these requirements are possible solutions for an individual briefing.

On the level of *handling* the most important requirement was: **The system should be easy to handle*.*** For some subjects the demo in the task-based part was already too difficult to use. *“Yeah, that would really have to be under guidance, yeah. [ …*] *So they [peers] wouldn’t be able to do it alone”* (CBP patient). This underlines the importance of the user-centered design, which is applied in the project through usability evaluations with the purpose of creating the most appealing and functional operation possible for the age group; if this is not possible, then use of the system under personal supervision must be considered.

The physiotherapists considered the requirement: **The system should offer breaks between exercises** to be the most important requirement in the level *duration of use*. Especially during home exercises, patients showed inconsistency in taking breaks. “*No, it then automatically follows that one stops or does not stop”* (CBP patient). Mostly the seniors in the groups performed the exercises all at once and without a break as a matter of course. Newcomers may be prone to overestimating themselves; especially in cases of chronic pain, over-ambitiousness can lead to severe pain. The patients should learn for their everyday life what individual dose of exercise is right for them and therefore they should have the opportunity to take a break. Another requirement the participants stated, was: **The exercises should last a maximum of 30 min**. Further a very important requirement was: **The system should offer 15 min of relaxation**. Psychoeducation plays an important role in multimodal pain therapy. The requirements analysis showed that relaxation therapy in VR can be offered in addition to movement training, but it should take less time in the game.

The physiotherapists considered the following requirement to be important in the level of *safety*: **The system should be used in a safe place with sufficient space.** Due to the lack of perception of the real space, many older adults can be exposed to the risk of falling. “*Of course, you must do such things [...] in a safe environment where there’s just enough room that you can’t knock anything over or put yourself in danger”* (CBP patient). This implies that a concept for safe use of the exergame is needed. **The system should allow you to sit down**. This approach also involves the risk of falling, which can result from overexertion, for example. A possibility of sitting or even executing the exercises in seated position would minimize the danger of falling. The following requirement was also given: **The system must contain a help button.**

In the level of financing the requirement: **The system should be offered for rent** was preferred. *“Renting would be a good solution.”* (CBP patient). This proposal would allow multiple users to share the system and make it available to a larger number of potential users.

### Hardware

This dimension did not consist of various levels. Due to the small number of requirements and semantically similar requirements, code groups were not created in the content analysis and were not presented in the prioritization as levels for this dimension. For this reason, this dimension consists of only one level. In the dimension hardware, the participants also identified the physiotherapists as the most important stakeholder. The psychotherapists were ranked 2nd and the CBP patients 3rd.

The physiotherapists determined the most important requirement to be the following: **The patients should be able to put on the googles by themselves**. This requirement is associated with another important requirement: **The goggles should be easy to put on and take off**. The ability to put the goggles on and take them off independently was perceived as important in order to enable the system to be used independently at home by older CBP patients. Here the hardware design must be adapted to the requirements of the seniors.

### Software

The software dimension consists of the following levels: *in-game environment, application of game* and *exercises*. In the dimension *software*, the participants identified the physiotherapists as the most important stakeholder. The psychotherapists were ranked 2nd and the CBP patients 3rd.

The decisive requirement in the level *in-game environment* was: **The system should perform an individual calibration (e.g. to detect movement limitations)**. The desire for calibration was mainly expressed by the physiotherapists. With an individually adapted environment, limitations of the patients could be integrated into the game. Patients are thus not forced by the game environment to make movements that they cannot functionally perform, e.g. due to a limited range of motion in a joint. *“Yes, but I could imagine, for example, that [...] the environment knows how big the patient is. Or how high he can stretch to get it calibrated”* (Physiotherapist). The physiotherapists stated that they could imagine, for example, a movement assessment in the VR to be carried out by the patient before the start of the game.

In the semantic level *application of game* the requirement: **The system should enable the therapist to intervene on patients (pain, anxiety, incorrect execution)** was prioritized in first place. This requirement of course depends on the setting in which the exergame is performed. If the exergame is performed in a setting in which the therapist stands next to the patient, immediate intervention is possible. In a setting where the patient performs exercises independently at home, a concept must be considered for the therapist’s intervention (e.g. though a help button/emergency-supporting system). *“Well, definitely intervene when there’s fear, when there’s irritation [...] can be a technical problem. [...] And of course even if I feel like someone can’t handle it, or do something completely different than (laughing) actually the model”* (Physiotherapist). The physiotherapists could consider an intervention, e.g. by assisting or stopping the game, if the patient experiences severe pain during the game, is afraid while playing, or performs the exercises incorrectly, which can lead to danger or increased back pain. During the task-based part, the phenomenon of cybersickness in a subject was detected once, but disappeared after a short break (approx. 5 min).

In the level of *exercise* the requirement: **The patients should perform everyday exercises** was preferred. The physiotherapists see the VR as a chance to try out tasks that are close to everyday life in a realistic way. *“In the training program one could use environments / tasks that are attractive for many older people or are close to everyday life – such as the garden ... planting something, harvesting or maybe shopping”* (physiotherapist)*.*

### Gamification / game integration

This dimension consists of the following levels: display of feedback, biofeedback, progress, and storytelling. In the dimension: gamification / game integration, the participants identified the psychotherapists as the most important stakeholder. The physiotherapists were ranked 2nd and the CBP patients 3rd.

At semantic level *display of feedback* the following requirement was perceived as the most important: **The feedback in the system should always reinforce positively, never negatively.** In the interviews, the physiotherapists also named this as an important method for giving feedback. This is particularly important in order to stabilize a long-term intrinsic motivation of CBP patients during the exercises in the exergame in order to support long-term training adherence.

**Fig. 3:**
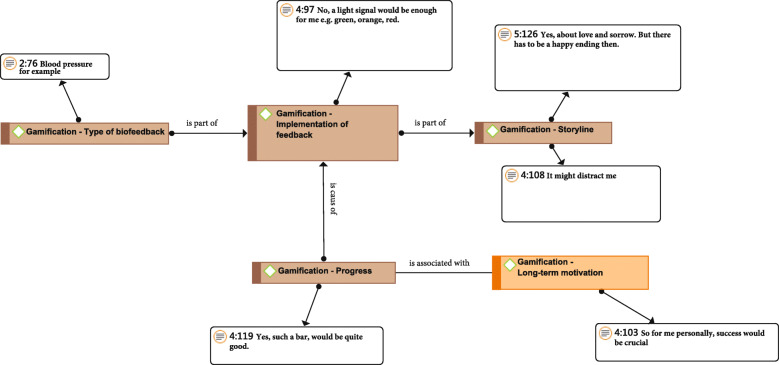
Relation network of gamification aspects in an adherence-increasing VR exergame for CBP pateients

In the level of *biofeedback* the participants considered the requirement: **The exergame is intended to provide the user with behavioral recommendations (e.g. advice on how to relax)** to be the most important requirement in the level of *biofeedback*. During the interviews the psychotherapists always expressed their views on psychoeducation in the exergame: *“Well education, I think, is of course very, very important in terms of stability of the spine for example, if there are fears, like: ‘that breaks everywhere and [...]’*” (Psychotherapists). In classical multimodal pain therapy, behavioral therapeutic aspects play an important role. These aspects should also find their place in an exergame for CBP patients. Particularly in forming habits, patients must be aware of why they perform the exercises and what can cause them harm. It is precisely through misconceptions that many patients develop fear avoidance beliefs or poor stress management. In order to avoid and counteract these negative strategies, the system can be supported by e.g. stress sensors that measure the pulse during training and to give positive feedback of vital parameters. By using quantitative measurable methods, behavioral recommendations can be made usable, comprehensible for the user, which contribute to therapy adherence (Fig. 3).

On the level of *progress* the most important requirement was: **The system should display praise and rewards (for example, text “Hooray”, “Congratulations”)**. The seniors often stated during the interviews that rewards after completing a task in the game would please them. *“Well, all right. A reward if you do the task or something. Like I said, I’m not the person who wants to win while playing. The thing itself has to be fun and it has to have something to do with creativity”* (CBP patient)*.* Figure 3 shows how progress is structured in the game and how it can lead to long-term motivation for the exercises, which is necessary in the case of chronic back pain. Since training progress is required with chronic patients, because of the poor exercise adherence and the risk doing exercise wrongly, the current training status (progress display) is needed in the game. In order to be able to control the progress of the patients in the exergame, feedback needs to be implemented in the game. This feedback has to be biofeedback, which records e.g. the movements or stress of the patients with sensors. The feedback used in the game should be also part of the storyline, if there is one, which would have a further motivating effect.

On the level of *storytelling* the most prioritized requirement was: **The system should not tell a continuous story**. The psychotherapists said that longer-term success would be possible through an appropriate individual division of work and recovery phases. This speaks against a strict story, as it is difficult to divide it up individually. Also some subjects said that a continuous story is not imaginable for them. *“I think that would kind of distract me. Then I would listen to the story and become inactive when I listen to it”* (CBP patients). On the other hand, five of the 10 test persons said that they could imagine a continuous story and would like to experience it in the VR.

## Discussion

Within the ViRST requirements analysis, we gathered mandatory requirements for the conception of a VR HMD-based active exergame for older users with chronic back pain.

The key requirements of the overall system showed a desire for an individual briefing for the system. In addition, a sufficient instruction is considered necessary not only by the CBP patients, but also by the therapists. These requirements call for a differentiated consideration of the target group of older people. Especially due to a strong divergence in the acceptance of technology, it is necessary to take a closer look at the existing skills and abilities of the target group. Wright’s study [28] showed that especially in geragogy, older adults are often nervous when it comes to independently mastering new technology. For this reason, it is important to take away the fear of new technologies in this vulnerable group if they are to use a VR exergame. Oesch et al. [29] showed in their study that self-regulated exercise using instruction leaflets were superior to exergames regarding adherence in a geriatric inpatient rehabilitation setting. However, this study was not tested with a VR system but with a Microsoft Kinect exergame, through the effect of immersion this result should be tested with a VR system, because different effects could occur.

The therapists recommended that the exercises should last a maximum of 30 min. This also matches the health and safety warning of the oculus [30]. They suggest taking at least a 10 to 15-min break every 30 min. It also corresponds to the conventional treatment time at the physiotherapist.

We determined in the requirements analysis also that a user-friendly handling of the system and hardware is important and can also contribute to an easier access for the peer group. In addition, older persons need expert training and instruction in the use of the system, especially with regard to immersion in VR and the risk of falls, which can be plausible with a wired VR HMD. For the user group, a wireless HMD would be the preferred option. The focus group with the physiotherapists confirmed, that the system should be used in a safe place with sufficient space and with appropriate safety regulations such as to allow the patient to sit down in VR or to use a help button. If the older users do not get along with the operation of the system, an instructor may be considered to be present during the exergame. Palazzo et al. [31] came to the result that the feeling of being supported by care providers is important in a home-based exercise program for patients with chronic low back pain. First and foremost, with the HMD, senior subjects cannot perceive dangers in the room, which exposes them to the risk of falling. In addition, cybersickness could occur that patients cannot deal with. If a VR game is to be implemented at the patient’s home, there must be a proven safety concept and the patient must know what to do in case of emergency. For this reason, further laboratory research is necessary in which patients can first test the system under supervision and the researchers can detect potential safety hazards during the use of VR HMDs by seniors.

Considering the use of augmented reality and its applications in comparison to VR systems, it is obvious that the handling AR involves a lower risk of falls [32, 33] but leads to other effects. Serious games with an AR approach for example can include physical-related interventions for motivating to do physical activities. In the AR virtual objects that coexist in the same space as the real world are displayed in addition to the real world for improved perception. Older adults, whose technical abilities in dealing with new technologies due to cognitive, physical, financial and social resources [34, 35] are lower than those of younger people, and whose generation did not grow up with digital technologies [36], could be overloaded by these effects [37]. Nevertheless, there is a lack of research not only in this respect. In general, less research has been done in the field of augmented reality in connection with pain therapy compared to VR [16, 38]. Besides, AR systems and their applications are more cost-intensive compared to VR. The application of AR for CBP patients who would like to train with a back pain program like ViRST in their own home would represent a major financial hurdle for them.

With regard to the financing of the system, a concept should also be considered for how the system could be affordable for the target group. In our requirement analysis the stakeholder preferred, that the system should be offered for rent. In view of the increasing poverty among the elderly in Germany [39] it is necessary for the price to exclude as few as possible. Obviously, this has to be considered differently for each country.

On the other hand, the physiotherapists stated, that virtual reality offers the opportunity to safely test everyday scenarios. The multimodal pain therapy experts suggest that exercises should be transferable to the everyday life of seniors. The requirement “the exergame is intended to provide the user with behavioral recommendations” was also raised in the requirement analysis for exergames by Paulino et al. [40]. Behavioral therapeutic aspects should also be integrated into an exergame for CBP patients. Valenzuela et al. [41] conducted a study for a home-based cognitive-motor step training displayed on a monitor for older adults. Our results show similarities to their qualitative main findings, which indicate that positive feedback on performance is important to increase self-efficacy for the peer group. The interviewees in our study confirmed that the feedback in the system should always reinforce positively, never negatively. In addition, the study also suggests that the system should be easy to handle to reduce barriers to the use of technologies. The study of Valenzuela et al. is in fact in the same age group, however it examines a different clinical condition and the exergame applied does not use a VR HMD, for this reason the openness of the subjects to use the technology at home must be questioned. In our study with CBP patients and a VR system we propose a proven safety concept as mentioned above if a VR game is implemented at the patient’s home.

The physiotherapist emphasized that the system should perform an individual calibration in order to detect movement limitations. From a technical point of view, it would be possible to use tracker, inertial measurement units (IMUs) or cameras for human pose estimation to detect the range of motion of the relevant joints of the patients and to adapt the objects in the VR. Stănică et al. also suggested to use movement tracking equipment and biosensors for training session configuration in VR systems in neurorehabilitation [42].

In psychoeducation a combination with biofeedback would be possible e.g. by a measurement of heart rate variability (HRV). This would be an easy way to measure stress and react appropriately in game situations to show seniors their stress level and to teach them how to change their behavior. As further biofeedback the controllers can be used and additionally trackers could be attached to the back of the patients to enable posture correction. Barmpoutis showed a way give haptic-based guidance in tele-therapy by using wearables with vibrator motors [43]. The biofeedback serves as a basis for gamification elements and the necessary feedback to achieve training progress. Through age-appropriate motivation, e.g. praise and rewards etc., the usage frequency of the exergame can increase, leading to guided training progress and long-term motivation and adherence, thereby relieving pain.

## Limitations

Nevertheless, the study had some limitations. An external limiting factor was the small number of participants. Due to a small and imbalanced number of experts, the results cannot be generalized easily. Furthermore, within the older participants, who all had no previous experience with VR, there was a lack of technical skills and a low understanding of the applied technology. However, an initial saturation of the results could be achieved. Furthermore, this study was a first step, as the current application was an exergame user experience prototype whereas a more specified application with a proven therapeutic benefit is expected to be developed in the course of the project. In this respect, the requirements must be tested and specified with older users in a VR back pain exergame with implemented gamification aspects.

## Conclusion

Within the scope of the requirements analysis 104 requirements could be identified, which represent the basis for the conception of an exergame and the use cases in the ongoing ViRST project to create a user-friendly VR exergame that motivates older chronic back pain patients to perform exercises regularly. If the exergame is a user-centred VR back pain training program, it is quite conceivable in the future to adapt it for other target groups and to transfer the system from the inpatient area to the domestic environment of the patients. Therefore, it should be ensured that the safety and motivational elements correspond to the expectations of the target group. A more effective multimodal pain therapy could be achieved by a higher adherence of the home-based exercises, which could reduce the long-term cost of the health system.

## Supplementary information

**Additional file 1.**

## Data Availability

The datasets generated are available from the corresponding author on reasonable request.

## Abbreviations

CPGQ: Chronic Pain Grade Questionnaire
CBP: Chronic back pain
VR: Virtual Reality
