# Supplementary material for: Virtual reality in pain therapy: a requirements analysis for older adults with chronic back pain
Source: J Neuroeng Rehabil. 2020 Sep 29;17:129. doi: 10.1186/s12984-020-00753-8 (PMC7523056; doi:10.1186/s12984-020-00753-8)
Supplement: Supplementary file 1 — Additional file 1. [file 12984_2020_753_MOESM1_ESM.pdf]

Full Title: Virtual Reality in Pain Therapy: A Requirements Analysis for Older Adults Users with Chronic Back Pain

Journal: Journal of NeuroEngineering and Rehabilitation
Article Type: Original Research

Corresponding Author: Oskar Stamm, M.Sc.
Institution: Charité Universitätsmedizin Berlin Berlin, GERMANY
E-mail address: oskar.stamm@charite.de

ViRST Prioritization of the requirements

The purpose of this document was to prioritize the requirements for the ViRST system (Virtual Reality for pain therapy) that have been raised by chronic back pain patients and experts (physiotherapists and psychotherapists). The following summary shows the results of the prioritization of 6 CBP patients, 3 physiotherapists and one psychotherapist.

The stakeholders were asked to answer the following questions by putting them in an order using **grades** (e.g. 1, 2, 3, 4 etc.) The easiest way is to assign the ranks one after the other. Thereby the number zero („0“) cannot be assigned to any order.

For Example:

1. Games for the ViRST-System

1.1 How do you evaluate the importance of the following groups of experts in relation to the games?

Physiotherapist	Psychotherapist	Pain patient
Rank: 1	Rank: 3	Rank: 2

1.2 Please rate the requirements according to your subjective importance through placing ranks.

1. The system should provide ball games.	Rank 4
2. The system should provide ball toss games.	Rank 2
3. The system should provide card games.	Rank 5
4. The system should provide board games.	Rank 3
5. The system should provide puzzle games.	Rank 1

Since there are only 5 requirements in the dimension „Games for the ViRST-System“, there are only 5 ranks to be assigned.

1. **How do you evaluate the importance of the following group of experts regarding the overall project on chronic back pain therapy using virtual reality?**

Results:

**Physiotherapists**		**Psychotherapists**		**Pain patients**	
Expert knowledge:	Joint replacement, Stroke, back pain, heart insufficiency, Spinal disc herniation, cervical spine / lumbar spine syndrome, functional impairment, meniscus wear and tear	Expert knowledge:	depressions, cognitive impairments, chronic pain, anxiety, psychosomatic complaints	Age:<br>Back pain duration:<br>Contraindications:<br>Limitation of balance:<br>Most severe pain in the last 3 months:	Ø = 75,9<br>> 6 Months<br>No<br>No<br>ØNRS = 6/10 (≙moderate Pain)
Work experience:	Employee	Work experience:	Head of a practice, Practice owner		
**Rank:** 1		**Rank:** 2		**Rank:** 2	

2. **ViRST overall system**

2.1 How do you evaluate the importance of the following stakeholders regarding the overall system?

Results:

Physiotherapists<br>Rank: 1	Psychotherapists<br>Rank: 2	Pain Patients<br>Rank: 3

2.2 Please evaluate the requirements according to your subjective importance through placing ranks.

Results:

Handling

The system should be easy to handle.	Rank	1
The system should be used standing up.	Rank	2
The system should be used in a seated position.	Rank	3
The system should not replace conventional therapy.	Rank	3
The system should only be offered to patients without cognitive impairment.	Rank	5
The system should be operated by hands.	Rank	6
The view should be stabilized when the glasses are shaking.	Rank	7
The system should only be offered for chronic patients without movement limitations.	Rank	8

Duration

The system should offer breaks between the exercises.	Rank 1
The exercises should last 30 minutes maximum.	Rank 2
The system should offer a 15 minute relaxation sequence.	Rank 3
The system should offer a 30 minute relaxation sequence.	Rank 4
The system **should not** offer breaks between the exercises	Rank 5

Instructions/Briefing

The system should offer an individual briefing.	Rank 1
The system should offer a tutorial for the right handling.	Rank 2
The briefing should be conducted by personal assistance.	Rank 3
The system should show the amount of correct motions respectively	Rank 4
The system should offer alternatives for exercises.	Rank 5
The patient has to be instructed.	Rank 6
The system should have an instruction manual.	Rank 7
The system should lead to a correct posture.	Rank 8
The performance with the system should take place under the supervision of	Rank 9
The exercises should be executed under a personal instruction.	Rank 10

Security/ Dataprotection

The system should be used at a safe place with sufficient space.	Rank 1
The system should allow one to sit down.	Rank 2
The system should have a help button.	Rank 3
The data security must be given.	Rank 4
The system should not involve twists during the exercises.	Rank 5

Price/ Funding

The system should be for rent.	Rank 1
The system should be up for loan.	Rank 2
The system should cost < 100€.	Rank 3
The system should cost < 50€.	Rank 4
The system should be purchased by a one time payment.	Rank 5
The System should cost < 500€.	Rank 6
The system should cost < 1000€.	Rank 7

3. **Hardware of the ViRST-Systems**

3.1 How do you evaluate the importance of the following group of experts regarding **the Hardware for the ViRST-System**?

Results:

Physiotherapists<br>Rank: 1	Psychotherapists<br>Rank: 2	Pain patient<br>Rank: 3

3.2 Please evaluate the requirements according to your subjective importance through placing ranks.

Results:

The patient should be able to put on the goggles by himself.	Rank 1
The goggles should be put on and off easily.	Rank 2
The controller should be lightweight.	Rank 3
The goggles should be lightweight.	Rank 4
The controller must lay in the hands conveniently.	Rank 5
The goggles should fit appropriately (without a chink of light and not too tight).	Rank 6
The foam of the goggles must be exchangeable due to hygiene reasons.	Rank 7
The goggles should have a manual focus adjustment.	Rank 8
The controller must be differentiable.	Rank 8

4. **Software of the ViRST-Systems**

4.1 Evaluation of the importance by the stakeholder regarding **the Software for the ViRST-System**

Results:

Physiotherapist<br>Rank: 1	Psychotherapist<br>Rank: 2	Pain patient<br>Rank: 3

4.2 Please evaluate the requirements according to your subjective importance through placing ranks.

Results:

Environment

The system should undertake an individual calibration (for the detection of restriction of motion).	Rank 1
The system should have a virtual instructor.	Rank 2
The System should offer a selectable environment (e.g. green meadow, colorful characters).	Rank 3
The virtual instructor should be a chronic back pain patient who describes his/her story of suffering and positive experiences.	Rank 4

The system should represent nature as an environment (e.g. forest, flower garden, water).	Rank 5
The system should offer a gymnasium as an environment.	Rank 6
The system should not stereotype.	Rank 7
The system is supposed to offer fairy tale environments.	Rank 8

Application

The system should enable the therapist to intervene on patients (pain, anxiety, misperformance).	Rank 1
If the scores become worse, the physiotherapist should intervene and include the errors in the conventional therapy.	Rank 2
The system should know the limitations / key figures of the patient (e.g. max. width of the stretch, height, weight).	Rank 3
The system should query the VAS scale (even at longer intervals).	Rank 3
The system shall perform a more detailed assessment after the VAS scale has been queried.	Rank 5
The software should give safety instructions.	Rank 6
The graphics of the software should be high-resolution.	Rank 7
The system should display players as avatars.	Rank 8

Exercise

The system should perform everyday exercises.	Rank 1
The system should contain aerobic exercises (for the perception of the body).	Rank 2
The system should offer mobilization exercises.	Rank 3
The system should display in the therapist view the duration of the exercises performed.	Rank 4
The system should offer environments for men (e.g. adventure) and women (e.g. flowers).	Rank 5
The system should **not** be used for everyday exercises.	Rank 6

5. Gamification / Game integration

5.1 How do you evaluate the importance of the following stakeholders regarding the **gamification**?

Results:

Physiotherapist<br>Rank: 2	Psychotherapist<br>Rank: 1	Pain patient<br>Rank: 3

5.2 Please evaluate the requirements according to your subjective importance through placing ranks.

Feedback

The feedback in the system should always strengthen positively, never negatively.	Rank 1
The system should display rewards (for example, points, score display).	Rank 2
The system should show hints on the correctness of the exercises (e.g. by showing a figure).	Rank 3
The system should indicate if a movement could cause harm.	Rank 4
The system should include tasks that require a balance of activity and recovery/relaxation.	Rank 5
The system should point out the incorrect execution of the exercises.	Rank 6
The system should contain an evaluation (e.g. number of exercises).	Rank 7
The feedback on the performance should be provided by a visual signal (green, orange, red).	Rank 8
The user should receive a prize as a reward (e.g. three diamonds).	Rank 9
The software should demonstrate the execution of the exercises with a verbal description.	Rank 10
The system should offer feedback in case of misperformance with a verbal description.	Rank 11
The system should display the number of correctly executed exercises.	Rank 12
The system should offer a visual signal as feedback.	Rank 13
The system should indicate the progress in points.	Rank 14
The system should display whether the duration of the training is sufficient.	Rank 15
The system should provide feedback on the exercises acoustically.	Rank 16
The system should display the time that has already been spent in virtual reality.	Rank 17
The system should follow medical recommendations as guidelines.	Rank 18
The system should classify the performances in standard ranges.	Rank 19
The system should have a humorous feedback.	Rank 20
The system should **not** indicate that the exercises have been performed incorrectly.	Rank 21

Biofeedback

The system should provide users with behavioral recommendations (e.g. advices on how to relax).	Rank 1
The system should **not** provide feedback through vibration.	Rank 2
The system should display the pulse.	Rank 3
The system should provide feedback through vibration.	Rank 4

Progress

The system should display praise and rewards (e.g. text "Hooray", "Congratulations").	Rank 1
The progress should be displayed with bars.	Rank 2
The system should display the final result after a session has been completed.	Rank 3
The system should contain performance levels / levels.	Rank 4
The system should increase the level of difficulty for a certain number of points.	Rank 5
The system should evaluate using a scoring system.	Rank 6
The system should indicate the progress in points.	Rank 7
The system should display a high score.	Rank 8
The system should contain tasks (e.g. treasure hunt).	Rank 9
If the system is used infrequently, the system should admonish the user.	Rank 10

Storytelling

The system should **not** tell a continuous story.	Rank 1
The system should motivate by personal address.	Rank 2
The story in the game should be presented separately from the exercises.	Rank 3
